# The Trojan Horse Within: Mechanisms of Immune Evasion in Breast Cancer

**DOI:** 10.47248/chp2603010001

**Published:** 2025-12-05

**Authors:** Biswajit Das, Charles W. Winterbottom, Shaheen S. Sikandar

**Affiliations:** 1. Department of Molecular, Cell and Developmental Biology, University of California - Santa Cruz, USA; 2. Genomics Institute, University of California - Santa Cruz, USA; 3. Institute for the Biology of Stem Cells, University of California - Santa Cruz, USA

**Keywords:** Breast cancer, Tumor heterogeneity, Tumor immune microenvironments, Immune evasion, Clinical trials

## Abstract

Breast cancer (BC) is the most common type of cancer among females, and the number of deaths due to BC has increased over the past few decades. BC is primarily categorized based on the receptor status of BC cells as hormone receptor-positive (HR+), human epidermal growth factor receptor 2-positive (HER2+), and triple-negative BC (TNBC). These subtypes differ significantly in their treatment strategies, prognosis, immunogenic nature, and response to immunotherapy. TNBC is the most aggressive with a poor prognosis, but a subset of TNBCs that express programmed cell death ligand 1, have shown promising responses to immune checkpoint inhibitors. Across BC subtypes, distinct immune cell subsets remain active in the tumor immune microenvironment (TIME) that either inhibit or promote the growth of cancer. In isolation, it is challenging for cancer cells to thrive in presence of the body’s immune system, however with the aid of other cells in the TIME, they can work together to evade immune detection by suppressing antigen presentation, modulating immune recognition markers, and recruiting immune-suppressive cells. In this review, we provide an overview of the BC immune evasion mechanisms and discuss aspects of immune evasion in relation to tumor heterogeneity and cellular plasticity. We also highlight successful clinical trials targeting immune-evasion markers and discuss the challenges and potential future directions for solving these problems.

## 1. Introduction

Globally, breast cancer (BC) continues to be the most commonly diagnosed disease in women; an estimated 2.3 million new cases were reported in 2020, and as per current projections, this number will rise to more than 3 million by 2040 [1–2]. Improvements in the diagnosis and treatment of BC over the past few decades have significantly improved patient outcomes, leading to a 43% decrease in mortality rates by 2020. However, current patterns suggest that progress is slowing down [3]. Despite different advancements in early cancer diagnosis and therapeutic strategies, metastatic BC remains a leading cause of death. This is mainly due to the tumor’s heterogeneous nature and its ability to resist conventional treatments [4]. BC can be classified molecularly into subtypes such as triple-negative, human epidermal growth factor receptor 2 positive (HER2+; encoded by *HER2*), and hormone receptor positive (HR+; luminal A and luminal B). Triple-negative BC (TNBC) has a high level of immunogenicity, but limited targeted therapy options and poor prognosis [5–7], underscoring the urgent necessity for the development of innovative therapeutic strategies [8].

The immune system has an intricate and dual impact on BC, as it is capable of both inhibiting and promoting tumor growth. This dynamic process, termed cancer immunoediting, consists of three separate phases: elimination, in which the immune system destroys cancer cells; equilibrium, in which cancer cells and immune responses coexist; and escape, in which cancer cells avoid immune detection and spread [9]. Innate immune cells, such as dendritic cells (DC) and natural killer (NK) cells, as well as adaptive immune cells like CD8^+^ cytotoxic T lymphocytes (CTL), cause acute inflammatory reactions in the early phases of tumor growth. These reactions either promote the death of tumor cells or select the clones of cancer cells that are resistant [10]. With the progression of chronic inflammation, the tumor immune microenvironment (TIME) becomes more suppressive in nature. Tumor-associated macrophages (TAMs), myeloid-derived suppressor cells (MDSCs), and regulatory T cells (Tregs) accumulate in the TIME. The expression of immunosuppressive cytokines, including transforming growth factor beta 1 (TGF-β1; encoded by *TGFB1*), and interleukin-10 (IL-10; encoded by *IL10*), together with immunoregulatory enzyme such as indoleamine 2,3-dioxygenase (IDO), as well as immune checkpoint molecules like programmed death-ligand 1 (PD-L1; encoded by *CD274*), cytotoxic T-lymphocyte-associated protein 4 (CTLA-4, encoded by *CTLA4*), and lymphocyte activating-3 (LAG-3; encoded by *LAG3*), additionally hampers the body’s ability to create an efficient immune response against cancer [11–13]. These alterations impede the process of antigen presentation, including reduced expression of major histocompatibility complex class I (MHC-I) molecules and T cell exhaustion (T_EX), thereby promoting the tumor’s ability to evade body immune detection [14,15]. Additionally, there are critical prognostic implications when tumor-infiltrating lymphocytes (TILs) are present, since higher TIL density is linked to better patient survival. This association is particularly significant in HER2+ and TNBC tumors [16,17].

Immunotherapy functions mainly by enhancing the body’s natural immune mechanisms to target and destroy cancer cells [18]. Certain types of cancers, such as melanoma, show a favorable response to immunotherapy and are thus classified as ‘hot’ tumors. Conversely, some cancers show poor responsiveness to immunotherapy and are referred to as ‘cold,’ ‘immune-privileged,’ or ‘immune-resistant’ tumors [19]. BC subtypes have variable immunogenicity, where TNBC is more likely to trigger an immune response [20,21]. In contrast, HER2+ BC often exhibit mechanisms that allow them to evade immune detection, resulting in lower efficacy of immunotherapeutic approaches in these cases [22,23]. Moreover, intrinsic heterogeneity in the tumor and cellular quiescence also contribute to immune evasion in BC [24,25]. Pembrolizumab is an immunotherapeutic medication that has been approved by the Food and Drug Administration to treat TNBC. However, clinical results show that this treatment only improves the therapeutic outcomes for a small percentage (15%–20%) of TNBC patients [26,27]. For instance, low levels of PD-L1 are seen in a subset of TNBC tumors, and this is linked to a decreased efficacy of PD-L1-targeting therapy [28].

In this review, we discuss the various immune components in the TIME and briefly outline the distinct immune profiles of BC, including tumor immune infiltration and its correlation with clinical outcomes. We also provide an overview of the molecular mechanisms underlying BC immune evasion, successful therapeutic approaches, challenges, and potential future directions.

## 2. The Immune Landscape in Breast Cancer

### 2.1. Different immune cells in the tumor-immune microenvironment

**Figure 1.**
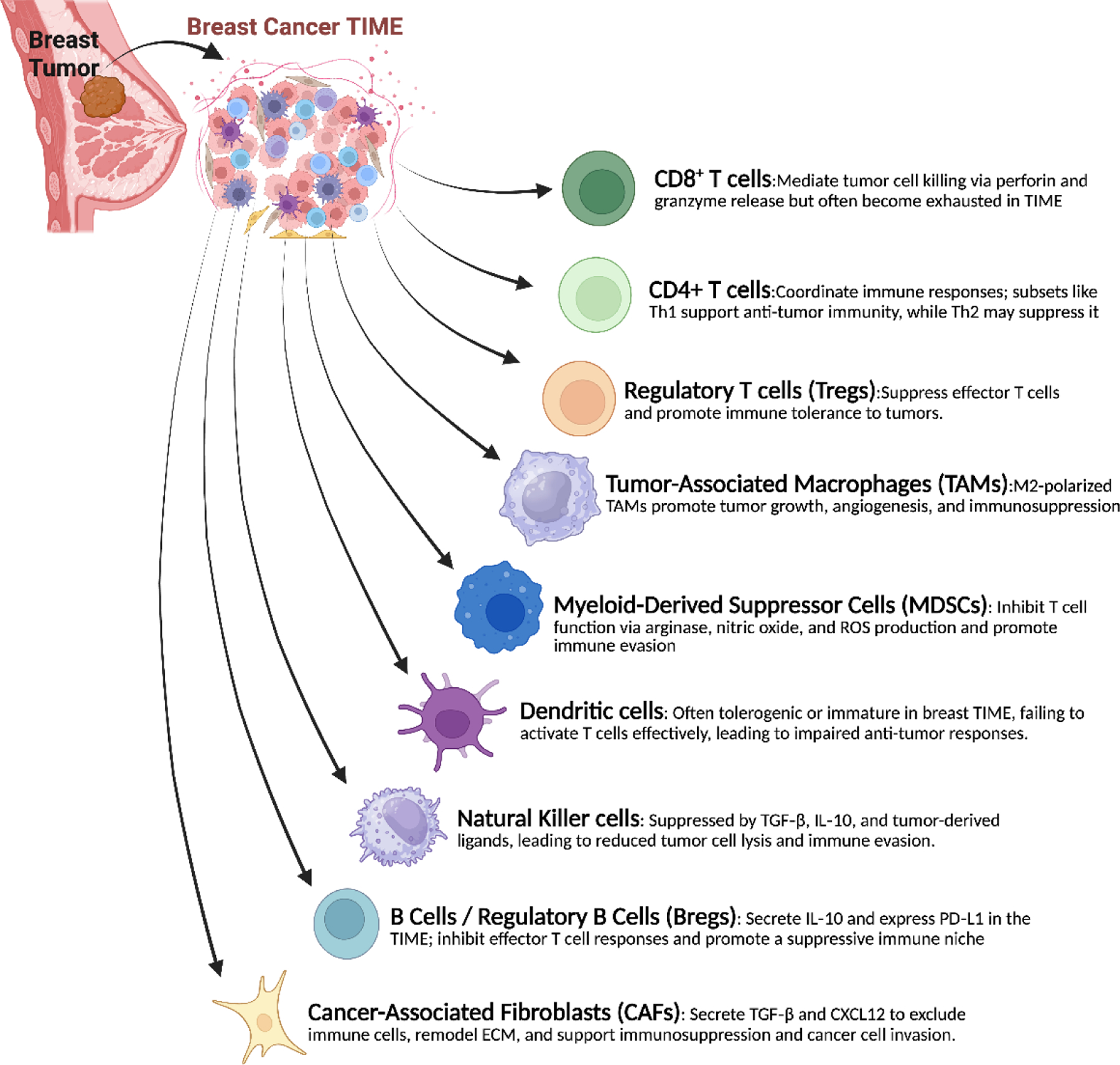
Different immune cells and their function in Tumor Immune Microenvironment (Created with BioRender.com).

In BC, the TIME plays a crucial role in determining the disease’s progression, immunological surveillance, and response to treatment. Cancer cells interact with it in intricate and dynamic ways through a variety of immune cell types. To improve successful immunotherapy methods, a thorough understanding of the makeup and functional roles of these immune components is essential. A brief explanation of their function is discussed in Figure 1.

#### T Cells:

T cells are essential for the immune system’s defense against malignancies. In particular, CTLs recognize antigenic peptides displayed on the surface by MHC-I molecules, which helps them locate and destroy tumor cells. The presence of these immune cells in breast tumors is associated with better patient outcomes and a greater chance of responding to immunotherapeutic treatments, especially in TNBC and HER2+ subtypes [29]. CD4^+^ Helper T (CD4^+^) cells are also essential for coordinating an effective immune response and for helping activate CTLs. However, the restrictive environment within the TIME and ongoing exposure to tumor antigens often hinder T cell activity in BC. This leads to T_EX, which is characterized by increased expression of inhibitory receptors such as CTLA-4 and programmed cell death protein-1 (PD-1; encoded by *PDCD1*) [30].

Tertiary Lymphoid Structures (TLSs) facilitate the recruitment and activation of CD4^+^ and CD8^+^ T cells in breast cancers, establishing distinct T-cell zones next to B-cell follicles. Chemokine gradients such as those established by C-C motif chemokine ligand 19 (CCL19; encoded by *CCL19*), C-C motif chemokine ligand 21 (CCL21; encoded by *CCL21*), and C-X-C motif chemokine ligand 13 (CXCL13; encoded by *CXCL13*) along with high endothelial venules within TLSs, promote T cell homing to the tumor, where antigen-presenting cells (dendritic cells and B cells) present tumor antigens to naïve T cells locally [31,32]. This local priming environment facilitates the differentiation of CD4^+^ T helper cells, particularly T follicular helper (Tfh) cells, and cytotoxic CD8^+^ T cells, without requiring migration to a remote lymph node. The presence of Tfh cells within TLSs in tumors has been shown to predict better clinical outcomes in breast cancer [32]. Tumors with more Tfh (CXCL13-producing) CD4^+^ cells had patients who lived longer and had higher rates of pathological complete response to therapy. Breast cancers with a lot of TLSs also have more CD8^+^ T cells, which often take on tissue-resident or stem-like phenotypes, and a gene expression profile that favors a type 1 CD4^+^ helper T cell (Th1)/cytotoxic immune response [32]. Notably, active TLSs usually have a higher ratio of effector T cells to regulatory T cells, and this TLS activity is linked to better prognosis [33]. Clinically, the presence of mature TLSs has been associated with improved immunotherapy efficacy: patients whose tumors exhibit abundant TLS with activated T cell infiltrates are more likely to respond positively to PD-1/PD-L1 checkpoint blockade [34–36]. Recent spatial transcriptomics studies in triple-negative cancers reveal that tumors with elevated TLS-associated T-cell infiltration frequently achieve complete eradication with anti-PD-1 therapy, whereas T cell-deficient TLS regions exhibit a less vigorous response until further interventions (e.g., radiation) stimulate additional T cells [34]. In summary, TLSs provide a structured setting for the recruitment, activation, and expansion of CD4^+^ and CD8^+^ T cells, thereby augmenting anti-tumor immune responses and improving the efficacy of immunotherapy in breast cancer.

#### Regulatory T Cells:

Tregs, a subgroup of CD4^+^ T cells identified by the transcription factor forkhead box p3 (FOXP3; encoded by *FOXP3*) expression, are essential for preserving immunological tolerance. These cells tend to proliferate in the TIME of BC, where they inhibit the immune system’s ability to fight the cancerous growth. Particularly in HER2+ BC subtypes, a higher percentage of tumor-infiltrating Tregs has been linked to worse patient outcomes and worse survival. Their primary mechanisms of immunosuppression include the release of cytokines such as TGF-β and IL-10, as well as signaling pathways regulated by CTLA-4 [37,38].

#### Tumor-Associated Macrophages:

Macrophages that infiltrate breast tumors, known as TAMs, can differentiate into either the M1 phenotype, which has pro-inflammatory properties, or the M2 phenotype, which exhibits anti-inflammatory functions [39,40]. In BC, the M2-like macrophage phenotype is the dominant form and plays a key role in supporting tumor growth, the development of new blood vessels, metastasis, and suppression of immune responses. These TAMs release cytokines such as IL-10, TGF-β, and vascular endothelial growth factor (VEGF), which hinder the activity of cytotoxic immune cells and promote tissue restructuring and vascular formation. The presence of M2-polarized TAMs is associated with higher tumor grades and worse patient outcomes [41,42].

#### Myeloid-Derived Suppressor Cells:

MDSCs constitute a diverse group of immature myeloid cells characterized by their potent immunosuppressive functions. These cells accumulate within the breast tumor microenvironment, where they inhibit T cell activation primarily through the secretion of enzymes and molecules such as arginase-1, inducible nitric oxide synthase (iNOS), and reactive oxygen species [43]. MDSCs impair the ability of NK cells to carry out their cytotoxic functions and promote the proliferation of Tregs, thereby establishing a highly immunosuppressive milieu [43].

#### Natural Killer Cells:

Innate lymphocytes known as NK cells can eliminate tumor cells directly without first encountering particular antigens. NK cell activity tends to decrease in the context of BC. Soluble substances produced in the TIME, such as prostaglandin E2 and TGF-β, contribute to this suppression by reducing the expression of activating receptors on NK cells. Furthermore, alterations in the expression of molecules associated with MHC-I molecules can hinder NK cells’ capacity to identify tumor cells. By selectively retaining or upregulating non-classical MHC-I molecules (e.g., human leukocyte antigen-E) that interact with inhibitory receptors, *i.e*., immune checkpoint molecular pair CD94 (encoded by *KLRD1*) and NKG2A (encoded by *KLRC1*), and simultaneously decreasing classical MHC-I expression, tumor cells inhibit NK-mediated cytotoxicity and promote immune evasion [44,45].

#### B Cells and Regulatory B Cells:

B lymphocytes play a complex role in BC. Through T cell activation, antigen presentation, and antibody production, they can support anti-tumor defenses. On the other hand, certain B cell subsets, referred to as regulatory B cells (Bregs), may aid in immune evasion by secreting TGF-β and IL-10, which suppress cytotoxic T cell activity and encourage the growth of regulatory T cells. There is still much to learn about the unique roles and characteristics of B cells in the tumor immune milieu [46].

Breast tumor-associated TLSs function as localized ‘mini-lymph nodes’ that attract and organize B cells through chemokine signals such as CXCL13, promoting the development of B cell follicles interconnected by follicular DC networks. In these structured niches, B cells undergo activation and clonal expansion, maturing into memory B cells and antibody-secreting plasma cells, similar to their development in germinal centers [31]. Importantly, TLS-resident B cells can serve as effective antigen-presenting cells that capture tumor antigens and deliver them to T cells, thereby initiating and enhancing localized T cell responses [31]. The existence of substantial B-cell-rich TLSs in breast tumors and other solid tumors is associated with positive patient outcomes; elevated TLS densities, frequently assessed through proliferating B-cell or high endothelial venule counts, correlate with prolonged survival and reduced recurrence rates. In one study in TNBC, numerous peritumoral TLSs served as an independent positive prognostic indicator, surpassing overall TIL counts in estimating relapse-free survival [31,35,36]. Recent single-cell studies show that there is a mix of B cells in TLSs, including follicular-like B cells that make tumor-specific IgG/IgA antibodies and are strongly linked to anti-tumor immunity and better immunotherapy effectiveness [47]. These findings collectively emphasize that TLSs create a specialized microenvironment conducive to B cell activation, plasma cell differentiation, and antigen presentation-processes that are collectively linked to improved breast cancer prognosis.

#### Dendritic Cells:

DCs are essential antigen-presenting cells that initiate anti-tumor immune responses and activate T cells. However, DCs in BC often have a phenotypic immaturity and a reduced ability to deliver antigens. Their maturation and functional abilities are hampered by factors generated by the tumor microenvironment, including VEGF and IL-10, which lead to poor T cell priming and the formation of immunological tolerance [48,49].

### 2.2. Dynamic remodeling of breast cancer TIME during tumor progression and therapies: Insights from single-cell and spatial transcriptomics

Recent single-cell investigations have significantly enhanced our understanding of the breast cancer immune microenvironment. For instance, Zhang *et al*. combined extensive single-cell RNA sequencing (scRNA-seq) data from TNBC patients undergoing chemotherapy (paclitaxel *versus* nab-paclitaxel) with or without PD-L1 blockade, revealing unique therapy-induced immune reprogramming. Nab-paclitaxel combined with anti-PD-L1 specifically increased T-cell factor 1-positive (TCF1^+^; encoded by *TCF7*) stem-like CD8^+^ T cells and CD4^+^ Tfh cells, while uniquely enriching myeloid subsets such as mast cells and pro-inflammatory macrophages, absent in conventional paclitaxel treatment [50]. This study highlighted mast cells as key orchestrators of anti-tumor immunity by promoting T and B cell recruitment and activation, and suggested that increasing mast cells could improve checkpoint immunotherapy [50]. Schalck *et al*. carried out single-cell RNA/DNA sequencing on paired biopsies from estrogen receptor-positive (ER+) breast tumors before and after neoadjuvant radiotherapy and found that the immune system changed quickly within a week of radiation. After radiotherapy, they found more ‘naive-like’ CD4^+^ T cells and newly activated CD8^+^ T cells coming into the tumor area, as well as the death of existing cytotoxic T cells and the arrival of myeloid cells [51]. Radiotherapy also caused different tumor clonal dynamics. Some tumors went through strong clonal selection, while others showed an interferon-induced phenotype with less clonal pruning [51]. As another example, spatially resolved single-cell profiling revealed 3 distinct immunological response trajectories in TNBC patients undergoing treatment with anti-PD-1 antibody (pembrolizumab) and radiotherapy. Tumors that didn’t respond, stayed ‘immune cold’, which means they did not have any TILs and did not change much after treatment. Tumors that did respond fell into two groups: one had pre-existing anti-tumor immunity with high MHCs and TLS at baseline, and the other was initially immunologically cold but later displayed a strong immune response (with a high number of cytotoxic T cell and antigen-presenting cells interactions) only after the combination of radio-immunotherapy [52].

Single-cell analyses show that TNBC has a uniquely immunosuppressive microenvironment compared to HR+ or HER2+ tumors [53]. TNBC tumors contain greater quantities of FOXP3+ regulatory T cells and exhausted CD8^+^ T cells (exhibiting increased dysfunction signatures), frequently accompanied by elevated plasma B cells. These characteristics indicate substantial immune infiltration in TNBC, albeit biased towards immunosuppressive phenotypes. On the other hand, HR+ and HER2+ breast cancers usually have fewer cytotoxic lymphocytes. For example, TNBC acquired more cytotoxic NK cells, while HER2+ and HR+ tumors do not have this trait [53]. Spatial transcriptomic profiling highlights intertumoral heterogeneity; for instance, an examination of 92 TNBC cases revealed 9 spatial ‘archetypes’ of tumor-stroma organization, with certain archetypes exhibiting dense TLS associated with enhanced prognosis and immunotherapy response [54]. Spatial mapping in HER2+ tumors identified areas of co-localized T cells and macrophages characterized by a type I interferon-rich signature and TLS-like structures, indicating that immunologically active niches can develop within HER2+ tumors [55]. TNBC and HER2+ subtypes are generally more immunogenic, frequently exhibiting elevated baseline TILs, in contrast to HR+ tumors, which are typically more ‘immune-cold’ [56]. These baseline differences in the TIME across subtypes affect cancer progression and therapy responses.

The breast undergoes significant remodeling as tumors transition from in situ to invasive and metastatic cancer. Single-cell and spatial transcriptomic studies demonstrate that during the progression from ductal carcinoma in situ (DCIS) to invasive ductal carcinoma (IDC), newly formed invasive tumors actively hijack immunosuppressive pathways. Cai *et al*. discovered that as DCIS develops to IDC, malignant cells develop increasingly aggressive transcriptomic profiles, while T cells transition into an exhausted state. Simultaneously, tumor cells upregulate various checkpoint ligands that interact with immune receptors to create an immunosuppressive niche [57]. Spatial analysis also validated the proximity of tumor cells to T cells/macrophages expressing these ligand-receptor pairs, demonstrating the reorganization of the *in situ* immune architecture to facilitate immune evasion [57]. After cancer becomes invasive, subsequent progression to metastasis is frequently linked with immune evasion. A single-cell RNA-seq analysis comparing primary and metastatic ER^+^ breast cancers revealed an enrichment of chemokine ligand 2-positive (CCL2^+^; encoded by *CCL2*) tumor-associated macrophages, FOXP3^+^ regulatory T cells, and exhausted CD8^+^ T cells in metastatic lesions. These metastatic sites exhibited significantly reduced productive tumor–immune interactions compared to primary tumors, resulting in a generally more immunosuppressive microenvironment [58]. Consistently, detailed atlases of metastatic breast cancer using multi-modal single-cell and spatial profiling have shown that some metastases have patterns of T cell exclusion, while others have localized T cell infiltration. These patterns are linked to the presence or absence of certain stromal programs in the area [56]. These studies show that breast tumors can change their immune environment as they grow. Early lesions may be kept in check by the immune system, but invasive growth and spread often happen at the same time as the immune system becomes less active and immune cells become less able to fight tumors.

Treatment significantly alters the TIME, and single cell/spatial studies are elucidating these dynamics. In neoadjuvant chemotherapy (NAC), baseline immune and stromal characteristics are crucial in assessing response, while the residual disease microenvironment varies by subtype. A study involving 100 patients with early breast cancer revealed that tumors exhibiting elevated epithelial-to-mesenchymal (EMT) gene signatures at diagnosis were less likely to attain a pathological complete response to neoadjuvant chemotherapy across all subtypes. Post-NAC residual tumors exhibited heightened EMT marker expression, particularly in residual HR+ cancers, indicating therapy-induced selection for mesenchymal, potentially immune-evasive tumor cells [59]. In terms of the immune system, pre-treatment TIL levels were linked to NAC outcomes in aggressive subtypes. For example, HER2+ tumors that responded had a lot more CD3^+^ lymphocytes at the initial stage than tumors that didn’t respond. Following chemotherapy, both HR+ and TNBC residual lesions demonstrated an elevation in CD8^+^ T cell infiltration relative to their pre-NAC condition, signifying an immune-reactive alteration in the tumor microenvironment post-therapy [59]. Recent single-cell profiling of HR+/HER2− metastatic breast cancer treated with cyclin-dependent kinase 4/6 (CDK4/6; encoded by *CDK4* and *CDK6*) inhibitors has revealed distinct TIME patterns correlated with therapeutic response within the framework of targeted therapy. Luo *et al*. noted that baseline tumors from patients who subsequently experienced prolonged progression-free survival during CDK4/6 blockade exhibited markedly elevated densities of infiltrating CD8^+^ T cells and NK cells compared to tumors from patients with rapid progression. This implies that an immunologically ‘hot’ microenvironment may enhance the effects of CDK4/6 inhibition [60]. Additionally, tumors from patients with late progression (those who initially responded but later developed resistance) exhibited upregulation of inflammatory pathways (e.g., c-Myc (MYC), EMT, and tumor necrosis factor (TNF-α; encoded by *TNF*) signaling pathways) and elevated immunosuppressive ligand-receptor interactions at relapse, such as secreted phosphoprotein 1 (SPP1; encoded by *SPP1*)-CD44 signaling, which can recruit suppressive macrophages when compared to baseline levels. Longitudinal biopsies validated dynamic alterations: in patients exhibiting later progression, NK cell proliferation and cytotoxic T cell activity intensified during therapy, although these effects were mitigated by increased expression of T cell inhibitory signals within the microenvironment [60].

Single-cell and spatial transcriptomic analysis of TNBC patients undergoing neoadjuvant pembrolizumab (anti–PD-1) treatment, with or without radiation, identified three distinct immune response patterns. Non-responders exhibited an ‘immune desert’ phenotype characterized by minimal T-cell infiltration both before and following therapy. Among the responders, one group showed a ‘hot’ immune microenvironment before the treatment. This was shown by a lot of CD8^+^ T cells, MHC class II^+^ antigen-presenting cells, and TLS. This group was likely to respond well to immune checkpoint blockade (ICB). The second responder group, which was initially immune-cold, only developed a strong immune response after receiving radiotherapy with increased cytotoxic T-cells and myeloid cells infiltration [52].

In summary, scRNA-seq and spatial transcriptomic methods have provided deeper insights into the tumor immune environment. Single-cell techniques can differentiate complex cellular states and dynamic subpopulations (e.g., stem-like cells, exhausted T cells, or specific macrophage subsets) that were previously indistinguishable through bulk flow cytometry or immunohistochemistry. Understanding these single-cell and spatially analyzed dynamics across breast cancer subtypes and treatment contexts is steering the advancement of more efficacious, tailored immunotherapy approaches in breast cancer.

### 2.3. Immune microenvironment of tumor initiating cells *vs*. differentiated cells

Tumor initiating cells (TICs) or cancer stem cells (CSC) inhabit a more immunosuppressive TIME than differentiated breast cancer cells. It is now well established that minority populations of TICs in the tumor display stem-like characteristics and have high cellular plasticity [61]. These cells are also quiescent and can remain dormant at metastatic sites. Indeed, a recent study demonstrated that the quiescent cancer within the tumor forms immune suppressive niches that resist T-cell attack and lead to T-cell exhaustion [62]. Moreover, hybrid cell states (epithelial-mesenchymal) that have high tumor-initiating capability have been shown to promote an immune suppressive niche and can protect the more differentiated cells in the tumor from ICB [63]. Depletion of specific factors such as CD73 on these hybrid cells can sensitize the tumor and metastatic colonization to anti-CTLA-4 ICB [64]. Interestingly, a study also showed that targeting of dormant disseminated tumor cells is essentially a matter of numbers. Because these rare cells evade immune surveillance due to their extremely low abundance, and by increasing the number of tumor-specific T-cells can lead to the elimination of residual metastatic BC cells [65].

TICs also actively shape their niche by recruiting regulatory immune cells and reducing their presentation of antigens. For example, TICs often have lower levels of MHC class I molecules, which makes them evade cytotoxic T lymphocytes. TICs also express the ‘don’t eat me’ signal, CD47, making them evade immune detection by macrophages [66,67]. They also have higher levels of immune checkpoint ligands like PD-L1. PD-L1 is up to 3-fold on TIC-enriched populations compared to differentiated tumor cells. This adaptive PD-L1 expression directly inhibits effector T cells and is driven by signaling pathways such as Notch3-mechanistic target of rapamycin (mTOR; encoded by *MTOR*) and phosphatidylinositol 3-kinase (PI3K; encoded by *PIK3CA*)/AKT serine/threonine kinase (AKT; encoded by *AKT1*), which also reinforce stemness [59,60]. In line with an ‘immune-privileged’ phenotype, tumors exhibiting elevated TIC marker expression frequently contain more immunosuppressive infiltrates, including FOXP3^+^ regulatory T cells (Tregs). TICs can actively convert conventional CD4^+^ T cells into Tregs; recent research demonstrated that TIC-derived exosomes containing the transcription factor FOXP3 can induce CD4^+^CD25^+^FOXP3^+^ Tregs within 24 hours [68]. TICs also release cytokines and factors (like TGF-β, IL-10, and CCL2) that change tumor-associated macrophages into M2 cells and attract MDSCs. This makes the TICs’ niche even more resistant to immune attack. For example, TICs secrete high levels of the cytokine macrophage migration inhibitory factor (MIF), which changes the TIME to immunosuppressive state. This leads to an increase in Tregs and suppressive neutrophils and a decrease in intratumoral CD8^+^ T cells. Silencing or inhibiting MIF notably reversed these effects, transforming macrophages into a proinflammatory phenotype and augmenting T cell infiltration [69]. These characteristics indicate that TICs can evade or inhibit anti-tumor immunity more efficiently than bulk tumor cells. In syngeneic mouse models, TICs exhibited increased resistance to T cell cytotoxicity [70]. On the other hand, their more differentiated counterparts, which have higher MHC-I and lower PD-L1, are easier for T cells to find and kill [71,72]. Remarkably, the immune response can increase the percentage of TICs in the tumor. For example, activated T cells make interferon-γ, which has been shown to reprogram non-stem breast cancer cells into new TIC. Additionally, checkpoint inhibitor therapy caused an increase in interferon gamma (IFNγ; encoded by *IFNG*), which amplifies classic TIC traits such as chemoresistance and metastasis [70]. Mechanistically, IFNγ-induced TIC plasticity was attributed to metabolic reprogramming e.g., via branched-chain amino acid transaminase 1 (BCAT1; encoded by *BCAT1*), which could be inhibited to enhance immunotherapy efficacy. Interestingly, breast cancer patients undergoing anti-PD-1/PD-L1 therapy have exhibited elevated expression of TIC-associated markers post-treatment, reflecting the findings observed in the *in vivo* mouse models [70]. This indicates that although immune checkpoint inhibitors (ICIs) activate T cells against the tumor mass, the residual TICs may resist immune eradication and potentially proliferate under immune pressure.

Responses to ICIs can thus vary between TICs and non-TICs. Differentiated tumor cells expressing PD-L1 can be eradicated when immune checkpoint inhibitors (e.g., anti-PD-1/PD-L1 antibodies) alleviate T cell suppression. TICs, on the other hand, are less likely to be affected by ICI-driven immunity because they have immune-evasive adaptations. TICs are resistant to immunotherapy, not only because PD-L1 inhibits T cells, but also because their low MHC-I expression and cytokine-rich suppressive niches protect them from being recognized and killed by T cells [73]. It is important to note that targeting the immune-evasive programs of TICs can enhance ICI responses. In TNBC models, neutralizing TIC-derived MIF resulted in a reduction of Tregs and an enhancement of CD8^+^ T cell infiltration in the TIME, significantly enhancing the efficacy of anti-PD-1 therapy. Similarly, blockade of PD-L1 or Notch pathways in TICs has been proposed to sensitize these cells to T cell attack [69,72]. In general, a complete treatment plan may be needed to stop TICs from escaping the immune system and improve long-term responses to immunotherapy. This could mean combining ICIs with drugs that target TICs or diminish their immunosuppressive niche.

### 2.4. Immune profiles across breast cancer subtypes

There are three main intrinsic subtypes of BC, (i) HR+, (ii) HER2+, and (iii) TNBC. The expression of immunological checkpoints, the degree of immune cell infiltration, and the reactions to immunotherapeutic interventions vary among these subtypes. Understanding these distinctions is essential in developing personalized immunotherapy strategies for every subtype.

#### 2.4.1. Hormone Receptor-Positive (HR+)/HER2− Negative Breast Cancer (HER2−)

The most common breast cancer subtypes express hormone receptors, including progesterone (PR) and estrogen (ER), and are classified as hormone receptor–positive, HER2-negative (HR+/HER2−) tumors, also known as luminal A and B subtypes. Compared to TNBC and HER2+ subtypes, these cancers typically exhibit lower levels of immune cell infiltration and react less favorably to immune-based treatments. They typically have fewer TILs, PD-L1 expression, and mutations, all of which add to their poor response to immune checkpoint inhibitors [7]. When detected, immune cell infiltration mainly involves cells that suppress immune activity, such as Tregs and M2 macrophages [29]. There is more immune cell infiltration in luminal B breast tumors than in luminal A cancers, which are typically more aggressive and exhibit higher levels of cell proliferation. However, improved treatment results are not necessarily the consequence of this enhanced immune activity [74]. It is difficult to target HR+ malignancies with immunotherapy because of their intrinsically ‘cold’ immune profile. The current focus of research is on using combination techniques, such as immune-stimulating drugs or epigenetic agents, to ‘heat up’ these tumors and increase their reactivity [75].

#### 2.4.2. HER2+ Breast Cancer

HER2 is overexpressed in HER2+ breast tumors, which account for 15%–25% of all cases of BC. This specific subtype has moderate immunogenicity and intermediate TIL levels, which have been demonstrated to predict improved responses to HER2−targeted treatments like pertuzumab and trastuzumab [76]. Recent studies indicate that the presence of TILs, especially CD8^+^ cytotoxic T cells, is associated with higher rates of achieving a complete pathological response following anti-HER2 treatment [77]. An important way that trastuzumab works as a treatment is through antibody-dependent cellular cytotoxicity (ADCC), which is triggered by immune effector cells. This emphasizes the critical role the immunological microenvironment plays in determining the treatment’s effectiveness [78]. Although at lesser levels than in TNBC, PD-L1 expression is also present in HER2+ malignancies. This suggests a possible, albeit diminished, susceptibility to immune checkpoint inhibitors. Clinical outcomes are generally better for HER2+ cancers with substantial immune cell infiltration, while HER2+ tumors with fewer TILs may be more likely to avoid immune identification [78].

#### 2.4.3. Triple-Negative Breast Cancer (TNBC)

The absence of HER2, progesterone receptor (PR), and estrogen receptor (ER) expression is a characteristic of TNBC. With the highest TIL density among the many kinds of BC, it is the most immunogenic subtype. A better prognosis is associated with TILs in TNBC, and their presence increases the tumor’s susceptibility to immune checkpoint blockade treatments and chemotherapy [79]. Immune checkpoint proteins like PD-L1 are often overexpressed in TNBC, which may boost the tumors’ susceptibility to immune therapies that target the PD-1/PD-L1 pathway [80]. TNBC has a higher mutational burden and neoantigen load, which further increases its immunogenicity [81]. A subset of patients with TNBC exhibits a positive response to immunotherapy, particularly when it is administered alongside chemotherapy [82]. Despite being immunogenic, TNBC can also develop adaptive resistance mechanisms or an immune evasion strategy, like recruiting Tregs or MDSCs to inhibit anti-tumor immune responses [9]. The comparative analysis of the immune infiltration profiles of the three intrinsic subtypes is presented below in Table 1.

### 2.5. Immune infiltration and clinical outcomes

The extent of immune cell infiltration in the breast tumor microenvironment, particularly by TILs, has emerged as an essential biomarker for prognosis and prediction in BC. Generally, higher TIL levels are associated with more favorable clinical outcomes, especially in TNBC and HER2+ subtypes. In cases of TNBC, increased TIL presence correlates with longer disease-free and overall survival. A comprehensive analysis involving over 3,700 patients demonstrated that for every 10% increase in stromal TILs, there is a significant reduction in the risk of cancer recurrence and death [79]. In HER2+ BC, increased infiltration of TILs is associated with a more favorable response to neoadjuvant anti-HER2 treatments such as trastuzumab and pertuzumab [76,77]. HR+ breast cancers typically exhibit low levels of TILs and are characterized by a less-inflamed tumor microenvironment. Consequently, these cancers tend to respond poorly to immunotherapy and are associated with a less favorable immune-related prognosis [29]. These findings underscore the value of immune infiltration as a prognostic biomarker and in guiding immunotherapeutic strategies.

## 3. Mechanisms of Immune Evasion in Breast Cancer

**Figure 2.**
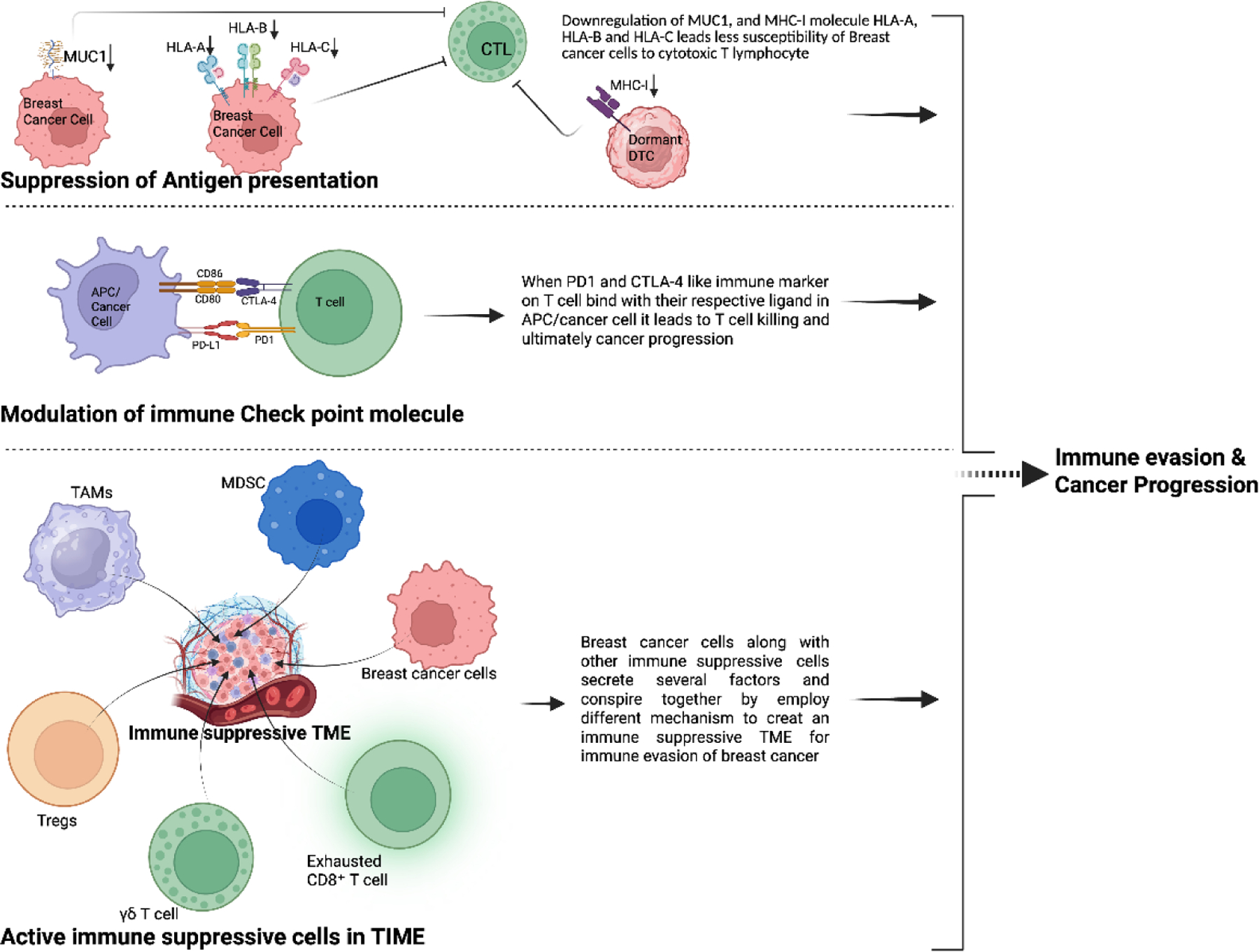
Overview of immune evasion mechanisms in breast cancer (Created with BioRender.com).

BC cells can display a range of mechanisms to suppress the body’s immune system and evade it. Since the body has its own active immune defense system, it is not always easy to hijack. Still, cancer cells manage to do so mainly through three central strategies: suppression of antigen presentation, modulation of immune checkpoint molecules, and creating an immunosuppressive environment in the TIME, mainly by recruiting different immunosuppressive cells. These strategies are explained below, and for easier understanding, a more straightforward pictorial presentation is shown in Figure 2.

### 3.1. Suppression of antigen presentation

The loss of tumor-associated antigens and the reduced expression of MHC class I molecules in BC are crucial strategies that enable tumors to evade immune detection, especially by CTLs [101]. In a study by Kontani *et al*., primary breast tumor samples were analyzed, showing that 70% of cases exhibited minimal destruction by the patient’s own CTLs, which was directly linked to decreased or altered expression of the mucin-1 (MUC1; encoded by *MUC1*) antigen. Notably, when MUC1 was reintroduced into a vaccine-resistant tumor cell line, the cells became susceptible to CTL-mediated killing again [102].

Epigenetic suppression of MHC-I is now seen as a key mechanism in the context of antigen presentation. Luo and colleagues showed that the DNA methyltransferase inhibitor guadecitabine effectively demethylates the promoters of MHC-I genes in human BC cell lines and mouse models [103]. Enhanced response to anti-PD-L1 immunotherapy, greater infiltration of CD8^+^ T-cells into the tumor, and normalization of MHC-I molecules on the cell surface were all results of this treatment, particularly when therapy was combined with IFN-γ [103].

MHC-I levels are considerably lower in a number of BC subtypes, according to recent research. According to Torigoe *et al*.’s research, immunohistochemical examination revealed that 85% of breast tumor samples (35 out of 41 cases) had reduced expression of HLA-ABC, a component of MHC-I. A decreased number of TILs was associated with this decrease in MHC-I expression, suggesting that tumor cells are less able to present antigens to CD8^+^ T cells, which may have an impact on immune evasion [104]. Recent research shows that dormant BC cells often lose MHC-I expression, making them less visible to CD8^+^ T lymphocytes. Their rarity and quiescent state reduce immune detection, allowing them to survive for long periods despite the presence of an active immune response [65].

In patients with advanced BC, studies have shown that peripheral DCs have a significantly diminished ability to present antigens, which results in decreased activation of T cells. When DCs are isolated from these patients, they induce a weaker response from allogeneic T cells, although the T cells themselves are still capable of functioning normally. Notably, DCs derived from hematopoietic precursors are able to restore typical T-cell stimulation, indicating that the problem resides in mature DCs rather than in the T cells directly [105,106]. Downregulation of classical MHC-I molecules (HLA-A, HLA-B, HLA-C) is commonly observed in BC, affecting approximately 77% of tumors, with complete loss seen in around 28%. This reduction is associated with decreased infiltration of lymphocytes into the tumor microenvironment, increased expression of hypoxia-inducible factor-1α (HIF-1α; encoded by *HIF1A*), and a poorer prognosis regarding distant metastasis-free survival [107–109]. A comprehensive review of several studies has shown that reduced HLA-I expression is associated with poorer disease-free survival rates (hazard ratio approximately 0.57). Nonetheless, its impact on overall survival has not been definitively established [110].

Moreover, proteomic analysis of TNBC samples showed that patients who experienced disease recurrence had notably reduced levels of several antigen-processing machinery components, such as Transporter Associated with Antigen Processing 1,2 (encoded by *TAP1* and *TAP2*), calreticulin (CALR), Human Leukocyte Antigen-A (HLA-A; encoded by *HLA-A*), endoplasmic reticulum aminopeptidase 1 (ERAP1; encoded by *ERAP1*), and tapasin (encoded by *TAPBP*). This decrease was strongly linked to a shorter period without recurrence and decreased overall survival, findings that were confirmed across various independent patient groups and publicly available gene expression datasets [111]. The loss of MHC-I expression can occur through epigenetic mechanisms that silence genes involved in antigen processing and presentation. In preclinical studies, using histone deacetylase (HDAC) inhibitors or DNA methyltransferase inhibitors has been shown to increase MHC-I levels, thereby enhancing the activation of cytotoxic T lymphocytes in BC [103]. Mal T-cell differentiation protein 2 (MAL2; encoded by *MAL2*), a protein involved in membrane raft formation and endosomal trafficking, influences the targeting of MHC-I molecules for degradation. Lowering MAL2 levels in TNBC cell lines increased the surface expression of MHC-I. This boost in MHC-I presentation enhanced CD8^+^ T-cell-mediated cytotoxicity both *in vitro* and *in vivo* [112]. The reduced expression of the phosphatase Protein Phosphatase 2 Regulatory Subunit B beta (PPP2R2B; encoded by *PPP2R2B*) in TNBC has been associated with impaired antigen processing and presentation and is also linked to a worse overall survival rate. *In vitro* studies have shown that PPP2R2B expression in TNBC cells can promote M1 polarization of macrophages and anti-tumorigenic responses [113].

Lastly, monocytes isolated from individuals with early-stage breast cancer show decreased expression of key markers, including intercellular adhesion molecule 1 (ICAM-1; encoded by *ICAM1*), CD80 & CD86, and lower levels of TNF-α. These changes impair their ability to present antigens effectively and lead to reduced stimulation of T-cell proliferation when exposed to common antigens like tetanus toxoid [114]. To find novel methods to increase antigen presentation, Zhu et.al, performed an organoid-based screen that stimulates antigen presentation and potentiates T-cell-mediated cytotoxicity. Previous studies show that epigenetic inhibitors against lysine demethylase 1A (LSD1; encoded by *KDM1A*), such as GSK-LSD1, CUDC-101, and the substance known as BML-210, increased expression of MHC-I. Moreover, using orthotopic models of breast cancer treatment with BML-210 substantially sensitized breast tumors to the inhibitor of the checkpoint programmed death-1 [115]. Strategies to increase antigen presentation can have significant implications in the treatment of BC and overcome the limitation of reduced immunogenicity.

### 3.2. Modulation of immune checkpoint molecules

#### 3.2.1. Programmed death-ligand 1 (PD-L1)

Programmed death-ligand 1 (PD-L1), an important regulatory molecule in immune response, is found to be increased in roughly 20 to 50 percent of breast cancer cases. The highest levels of PD-L1 expression are observed in more aggressive subtypes, such as TNBC [116]. The PD-1/PD-L1 ligand-receptor interaction leads to the recruitment of SH2-domain-containing protein tyrosine phosphatase 2 (SHP-2; encoded by *PTPN11*), which subsequently dephosphorylates T-cell receptor (TCR)-associated complexes. This process can inhibit CTL proliferation and cytokine secretion, ultimately reducing antitumor immune responses [117]. Interestingly, BC stem-like cells that display high cellular plasticity exhibit up to a threefold increase in PD-L1 expression compared to their non-stem cell counterparts through the Notch3/PI3K-AKT/mTOR signaling pathway [72].

Moreover, loss of the phosphatase and tensin homolog (PTEN; encoded by *PTEN*), through activation of the PI3K/AKT pathway, stimulates the transcription of PD-L1 as shown in TNBC cell lines such as MDA-MB-231. This correlates with decreased proliferation of CD8^+^ T cells and increased apoptosis of these immune cells. Clinically, TNBC tumors that are PD-L1 positive tend to have higher infiltration of CD8^+^ T cells but demonstrate impaired T cell effector functions, highlighting an immune environment that is inflamed yet immunosuppressed [116]. Furthermore, cytokines within the tumor microenvironment, especially IFN-γ, which signals through the Janus kinase/signal transducer and activator of transcription 1/interferon regulatory factor 1 (JAK/STAT1/IRF1; encoded by *JAK, STAT1*, and *IRF1*) pathway, and interleukin-6 (IL-6; encoded by *IL6*), which signals through the JAK/signal transducer and activator of transcription 3 (STAT3; encoded by *STAT3*) pathway, induce the expression of PD-L1. This process establishes a feedback loop that promotes tumor immune evasion [118]. Breast cancer cells also utilize oncogenic drivers like MUC1-C and MYC, which in turn activate nuclear factor-kappa beta (NF-κB; encoded by the *NFKB* family of genes) signaling pathways. This activation leads to an increased transcription and stabilization of PD-L1, contributing to immune evasion [119].

PD-L1 expressed on breast cancer cells interacts with PD-1 receptors on CTLs, leading to the suppression of CTL activation, proliferation, and cytokine secretion. Studies using mouse models have demonstrated that tumor-derived PD-L1 alone can effectively inhibit CTL-mediated killing and facilitate tumor immune evasion [120]. Additionally, particularly those exhibiting an M2-polarized phenotype, commonly express PD-L1. This expression plays a significant role in promoting local immunosuppression [121]. Breast cancer-derived small extracellular vesicles (sEVs) containing microRNAs such as miR-106b-5p and miR-18a-5p induces the upregulation of PD-L1 on TAMs by activating PTEN and protein inhibitor of activated STAT 3 (encoded by PIAS3)/STAT3 signaling pathways, thereby promoting metastatic progression [122]. Factors such as Annexin A1 (ANXA1; encoded by ANXA1), adapter molecule Crk (Crk; encoded by *CRK*), Cyclin-dependent kinase 8 (CDK8; encoded by CDK8), Guanylate-binding protein 5 (GBP5; encoded by *GBP5*), and Tumor necrosis factor receptor 2 (TNFR2; encoded by *TNFRSF1B*), play a role in increasing PDL1 expression, thereby facilitating immune evasion in breast cancer. These proteins contribute to the complex regulatory network that enables tumor cells to evade immune detection in breast cancer [123]. A recent study showed that in TNBC, the loss of Zinc Finger Protein 652 (ZNF652; encoded by ZNF652) removes the repression of PD-L1 gene expression. This results in increased levels of PD-L1, which helps the tumor evade the immune system by preventing T cell activation in the tumor microenvironment. Restoring ZNF652 reduces PD-L1 levels and enhances the body’s anti-tumor immune response [124]. Furthermore, Niclosamide prevents the RNA-binding protein human antigen R (HuR; encoded by *ELAV1*) from stabilizing PD-L1 mRNA in triple-negative breast cancer, leading to decreased PD-L1 glycosylation and expression. This reactivates T cell activity and improves the effectiveness of anti-PD-1 immunotherapy in mouse breast tumor models, identifying HuR as a new immune evasion target [125].

##### Therapy-Induced PD-L1 Regulation on Tumor Cells:

Chemotherapeutic agents can induce PD-L1 upregulation in tumor cells via various regulatory mechanisms. Chemotherapy-induced genotoxic stress frequently triggers transcriptional programs (e.g., through NF-κB or interferon signaling) that enhance PD-L1 expression. For example, breast tumors that survive chemotherapy exhibit an interferon-response gene signature characterized by elevated PD-L1 levels, resulting from chemotherapy-induced chromatin modifications that facilitate the recruitment of the IRF1 transcription factor to the PD-L1 gene promoter [126]. At the same time, stress pathways activated by chemotherapy (like NF-κB activation by oncogenic signals) can boost both PD-L1 gene transcription and PD-L1 protein stabilization, which increases the amount of PD-L1 on the cell surface. Cytotoxic chemotherapy can also induce cellular senescence in tumor cells, resulting in significant immune consequences. Senescent tumor cells produce a pro-inflammatory secretome, senescence-associated secretory phenotype (SASP), that attracts immune cells. In a model of breast cancer brain metastasis, doxorubicin-induced senescent cells specifically recruited PD-1-expressing T cells to the tumor, enhancing the effectiveness of anti-PD-1 immunotherapy in a CD8^+^ T cell–dependent manner [127]. Residual tumors that have been treated with chemotherapy often upregulate several immune checkpoint ligands to escape treatment. Recent research has delineated two predominant subpopulations of chemotherapy-resistant senescent tumor cells: one subset enhances PD-L1 expression through an IFNγ/IRF1-mediated pathway, while another subset elevates CD80 expression via a p53-dependent mechanism [126]. This unnecessary induction of PD-L1 and other checkpoints in tumors treated with chemotherapy makes immunotherapy even harder. Blocking one inhibitory ligand (like PD-L1) might not be enough because other checkpoints (like CD80 and others) also help the tumor avoid the immune system at the same time [126].

Cyclin-dependent kinase 4/6 inhibitors (CDK4/6i), utilized in breast cancer treatment, also exert considerable influence on tumor-cell PD-L1 regulation and the immune microenvironment. Inhibiting CDK4/6 not only stops the cell cycle but also boosts the body’s ability to fight cancer by changing the immune checkpoints that are built into the tumor. Preclinical studies demonstrate that CDK4/6i treatment markedly enhances the efficacy of PD-1/PD-L1 checkpoint inhibitors, primarily by augmenting the immunogenicity of tumor cells [128]. Blocking CDK4/6 can make tumor cells produce more MHC class I and PD-L1 and more chemokines that attract T cells. This leads to a tumor microenvironment that is inflamed by T cells, with more cytotoxic T lymphocytes getting into the tumor, which synergizes with PD-1/PD-L1 antibodies to give better tumor suppression. For instance, Goel *et al*.’s groundbreaking study showed that blocking CDK4/6 triggers anti-tumor immunity in breast cancer models, resulting in strong CD8^+^ T-cell infiltration and better tumor rejection when combined with PD-L1 blockade [128,129]. Mechanistically, CDK4/6 inhibitors can modify the tumor’s epigenetic landscape to promote immune activation. Inhibition of CDK4/6 has been demonstrated to cause extensive chromatin enhancer remodeling, including the activation of enhancers adjacent to interferon-stimulated genes, via the upregulation of activator protein 1 (AP-1) family transcription factors [130]. This AP-1-driven enhancer reprogramming increases the expression of several immune-regulatory genes, which could involve PD-L1, which further supports an immune-supportive environment [130].

Recent discoveries indicate that chemotherapy-induced senescence facilitates immune evasion by post-translationally stabilizing the PD-L1 protein. In senescent tumor cells, the upregulation of ribophorin 1 (RPN1; encoded by *RPN1*) enhances the N-linked glycosylation of PD-L1, thereby preserving it from degradation and increasing its surface expression, irrespective of continuous transcriptional activity. This ongoing PD-L1 ‘shield’ weakens the cytotoxicity of T cells, which allows senescent tumor cells to evade the immune system and possibly resist immune checkpoint blockade. Interestingly, RPN1 levels positively correlate with PD-L1 levels in several cancers, including breast cancer [131]. These findings emphasize a therapy-induced mechanism of immune escape with implications for combination strategies targeting PD-L1 stability.

#### 3.2.2. Cytotoxic T-Lymphocyte Antigen-4 (CTLA-4)

CTLA-4, is an immune checkpoint receptor on T cells that reduces their activation. It binds to CD80 or CD86 on antigen-presenting cells, blocking the stimulation of T cells and weakening the immune response [132]. It is frequently overexpressed in breast tumors, detected in ~53% of invasive breast cancers, while absent in normal breast tissue [133]. Elevated CTLA-4 expression on breast cancer cells locally correlates with increased TILs and, counterintuitively, with improved clinical outcomes in multiple cohorts, particularly in HER2+ and basal-like subtypes. High levels of CTLA-4 expression, exceeding the 63rd percentile, are linked to very low rates of recurrence and breast cancer-related deaths in the Oslo1, SCAN-B, and METABRIC groups [134]. CTLA-4, which inhibits immune responses, actually indicates an active immune response. CTLA-4 levels are low in inactive T cells but surge when T cells become activated. This rapid increase is crucial for the immune system to maintain balance. Therefore, the presence of this inhibitory signal on breast cancer cells is a sign that the immune system is actively engaged. CTLA-4 suppresses dendritic cell maturation and function by activating the extracellular signal–regulated kinase (ERK; encoded by MAPK1) and STAT3 pathways, which impairs T cell activation and anti-tumor immunity. Therefore, blocking CTLA-4 restores DC function, enhances T cell responses, and induces cancer cell apoptosis, presenting CTLA-4 as a therapeutic target in BC [135].

CTLA-4 is upregulated on activated CD4^+^ and CD8^+^ T cells as well as on Tregs, where it outcompetes CD28 for CD80/CD86 binding. This not only inhibits effector T cell activation—reducing interleukin 2 (IL-2; encoded by *IL2*), IFN-γ, and cyclin production—but it also endows Tregs with suppressive functions through ligand removal via trans-endocytosis [136]. Breast cancer cells utilize CTLA-4 to physically remove CD80/CD86 from antigen-presenting cells via force-mediated trans-endocytosis. This process depletes co-stimulatory ligands, drastically reducing antigen-presenting cell (APC) capacity to activate T cells [137]. Upon ligand binding on DCs and other APCs, CTLA-4 induces indoleamine 2,3-dioxygenase enzyme (IDO; encoded by *IDO1*) expression and TGF-β secretion—creating a tolerogenic microenvironment by depleting tryptophan and promoting regulatory Treg differentiation [138]. Similar to PDL1, CTLA4 is also regulated by many other factors such as peroxidasin-like protein (encoded by *PXDNL*), galanin (GAL; encoded by *GAL*), TNFR2, MYC, and ICAM1 [123]. In breast cancer, CTLA-4 is highly co-expressed with PD-1, LAG-3, T-cell immunoglobulin and mucin-domain-containing-3 (TIM-3; encoded by *HAVCR2*), and T Cell Immunoreceptor With Ig And ITIM Domains (TIGIT; encoded by *TIGIT*) on tumor-infiltrating lymphocytes, suggesting that CTLA-4 blockade may synergize with other checkpoint inhibitors [139].

#### 3.2.3. Other immune checkpoints molecules

B7-H3 (encoded by *CD276*) and B7-H4 (encoded by *VTCN1*) are newly recognized co-inhibitory molecules in the B7 family, playing a role in helping tumors escape the immune response in TNBC. However, their exact receptors remain largely unidentified. B7-H3, which is historically considered an orphan ligand, is present on APCs, CTLs, NK cells, and tumor cells. In TNBC, it is especially prevalent in TAMs, where its high levels are strongly linked to increased metastasis and worse patient outcomes [140]. The rise in B7-H3 levels creates an environment within the tumor that prevents the immune system from attacking it. Preclinical studies have shown that using targeted antibodies to block B7-H3 can improve the effectiveness of other immune-based treatments, such as anti-PD-L1 therapies [141,142]. Similarly, B7-H4, another member of the same family, is known to promote EMT, which further enhances the immunosuppressive TIME in TNBC [143–145].

The TIM-3 signaling pathway is another key immune checkpoint. TIM-3 is a transmembrane protein found on various immune cells, including CTLs, monocytes, macrophages, NK cells, and dendritic cells. It plays a role in sending inhibitory signals that lead to T cell exhaustion and contribute to immune tolerance. Initially considered an orphan receptor, TIM-3 was later found to interact with carcinoembryonic antigen-related cell adhesion molecule 1 (CEACAM1; encoded by *CEACAM1*), which is expressed on activated T cells and helps mediate inhibitory signals when it binds to TIM-3 [146].

Galectin-9 (GAL-9; encoded by *LGALS9*) acts as an important ligand for TIM-3, involved in suppressing CD4^+^ T helper cell activity. Notably, the levels of both TIM-3 and GAL-9 rise after anti-PD-1/PD-L1 treatment in TNBC, suggesting their potential role in developing resistance to therapy [147–149]. Supporting this, the gene *LGALS2*, which encodes Galectin-2, has been linked to greater infiltration of TIM-3+ CTLs within the tumor microenvironment, suggesting a more extensive role of galectin in modulating TIM-3 signaling [150].

LAG-3 is expressed at higher levels on both effector and regulatory T cells within the tumor microenvironment. It interacts with MHC class II molecules, which leads to decreased activation of T cells and enhanced suppressive activity of Tregs [151]. In breast cancer cohorts, LAG-3 expression is associated with markers of T-cell exhaustion and co-inhibitory molecules such as TIGIT and PD-L1, indicating its potential role in regulating T-cell dysfunction and impairing anti-tumor immune responses [152]. Clinically, combination therapies such as the LAG-3 fusion protein eftilagimod alpha, in combination with taxane-based chemotherapy, have shown promising results in activating the immune response and achieving favorable response rates in patients with metastatic breast cancer [153].

TIGIT, mainly expressed on exhausted CD8^+^ T cells, Tregs, and NK cells, interacts with a specific ligand on tumor cells, transmitting inhibitory signals through Immunoreceptor Tyrosine-based Inhibitory Motifs (ITIM) and Immunoreceptor Tyrosine-based Transferase (ITT) motifs [154]. In cases of TNBC, the expression levels of TIGIT are increased. This elevation is closely associated with altered tumor metabolism and impaired CD8^+^ T cell function, primarily through activation of the PI3K/AKT/mTOR signaling pathway [147]. Inhibiting TIGIT enhances the functional activity of CD8^+^ T cells both *in vitro* and *in vivo*. Additionally, early-stage experimental models indicate that combining TIGIT blockade with PD-1 or PD-L1 inhibitors produces a synergistic effect, resulting in improved anti-tumor responses [155].

V-domain Ig suppressor of T cell activation (VISTA; encoded by *VSIR*), a checkpoint molecule expressed on naive CD4^+^ T cells, Tregs, and myeloid cell subsets, plays a multifaceted role in the context of breast cancer [156,157]. The expression level is elevated in TNBC tumors characterized by a high density of stromal TILs. This elevation correlates with an immunostimulatory polarization of macrophages towards the M1 phenotype and is associated with a better patient prognosis [158]. Blocking aldehyde dehydrogenase 2 (ALDH-2) enhances the cytotoxic function of CD8^+^ T cells against tumors by inhibiting the expression of VISTA, a protein that facilitates tumor immune evasion [159]. Although clinical inhibitors are still in the early stages of development, dual targeting of VISTA and PD-L1 (such as CA-170) has demonstrated encouraging results in treating various solid tumors [160].

### 3.3. Immunosuppressive niche and cells in the tumor microenvironment

BC progression is significantly affected by the TIME, which contains BC cells and different immune cells that tend to inhibit the body’s anti-tumor defenses. BC cells across all subtypes (ER^+^, HER2^+^, TNBC) secrete various factors such as TGF-β, IL-10, CCL2 & C-C motif chemokine ligand 5 (CCL5; encoded by *CCL5*), colony-stimulating factors, prostaglandin E_2_, and indoleamine 2,3-Dioxygenase, that prevent anti-tumor immunity and create an immunosuppressive environment [161–165]. On the other hand, after the recruitment of specific immune suppressive cells like TAMs, MDSCs, Tregs, a specific subtype of γδ T cells producing interleukin-17 (IL-17; encoded by the *IL-17* family), and exhausted CD8^+^ T cells have all become significant factors in helping the tumor evade the immune system.

#### 3.3.1. Tumor-associated macrophages

TAMs, especially those that take on an M2-like role (also called CD163), play a key part in suppressing the immune response in the breast cancer tumor environment. These M2 TAMs release large amounts of immune-modulating substances like IL-10, TGF-β, and prostaglandin E2, and they also express PD-L1. They produce enzymes like arginase-1 and ROS, and they help attract Tregs. All these actions together hinder the ability of CD8^+^ T cells to activate, weaken NK cell killing, and impair dendritic cells’ capacity to present antigens. In growing tumors, cancer cells reprogram TAMs to support tumor progression by secreting immune-suppressing cytokines, activating checkpoint pathways, promoting new blood vessel growth, and remodeling the surrounding tissue [166].

Clinically, elevated TAM infiltration, especially high CD163^+^ TAM density, is strongly linked to poor outcomes. A meta-analysis of 8,496 breast cancer patients across 32 studies found that increased CD68^+^ and CD163^+^ TAM densities are significantly associated with decreased overall survival (HR= 1.7–2.5) and shorter disease-free survival (HR=1.8) [103]. In HER2^+^ breast cancer, high CD163^+^ TAM counts predicted worse survival regardless of trastuzumab use, emphasizing the immunosuppressive role of M2-macrophages in therapy-resistant cases [167].

scRNA-seq studies have revealed significant TAM heterogeneity in breast cancer. One atlas study of over 49,000 TAMs identified multiple subtypes with different functions: some help regulate adaptive immunity through T cell interactions, while others promote angiogenesis and lymphangiogenesis via endothelial cell crosstalk [168]. Another integrative analysis of scRNA-seq and bulk RNA-seq identified a new SPP1^+^ and complement component 1, q subcomponent, alpha polypeptide-positive (C1QA^+^; encoded by *C1QA*) macrophage subtype linked to poor prognosis and chemotherapy resistance, emphasizing functional differences among TAMs [169]. TAMs, especially the M2-like type marked by CD163^+^, help the cancer avoid the immune system. They achieve this by dampening the activity of cancer-killing immune cells, impairing the immune system’s ability to detect the tumor, and recruiting immune cells that actively suppress the anti-tumor response. Higher numbers of TAMs are usually linked to a poorer outlook and resistance to treatment [170,171].

#### 3.3.2. T regulatory cells

Tregs, identified by their expression of CD4, CD25, and FOXP3, serve as key suppressors of anti-tumor immune responses in the breast cancer TIME [15]. Higher levels of FOXP3^+^ Tregs are linked to more aggressive tumors and worse patient outcomes. A review of 14 studies with over 10,000 breast cancer patients found that having a lot of Tregs is strongly associated with higher tumor grades, lack of ER positivity, HER2 positivity, and a greater chance of cancer relapse [172].

Functionally, Tregs mediate immune suppression through various mechanisms. They secrete immunosuppressive cytokines, such as IL-10 and TGF-β, inhibit dendritic cell maturation, and suppress the proliferation of effector T cells [173]. Moreover, Tregs express CTLA-4, which downregulates CD80/CD86 on antigen-presenting cells and induces PD-L1 expression, thereby promoting local immune response tolerance [174]. The recruitment of Tregs to the tumor site is driven by the chemokine C-C motif chemokine ligand 22 (CCL22; encoded by *CCL22*), which is secreted by tumor-associated macrophages and tumor cells. This chemokine attracts C-C motif chemokine receptor 4-positive (CCR4^+^; encoded by *CCR4*) Tregs into the TME, where they proliferate and exert suppressive effects [175,176].

High levels of chemokine receptor C-C motif chemokine receptor 8 (CCR8; encoded by *CCR8*) are linked to poor prognosis in breast cancer. Treg cells express a high amount of CCR8 and promote breast cancer progression [177]. In murine breast cancer models, depletion of Tregs using anti-CD25 antibodies or genetic ablation (FoxP3-Diphtheria Toxin system) enhances CD8^+^ T cell infiltration and delays tumor progression, confirming their direct role in suppressing anti-tumor immunity [178].

#### 3.3.3. Exhausted CD8^+^ T Cells

In breast cancer, particularly in ER^+^ and TNBC subtypes, chronic exposure to antigens and ongoing inflammation associated with the tumor promote the transformation of CD8^+^ T cells into an exhausted state. These exhausted T cells show high levels of inhibitory receptors such as PD-1, TIM-3, and LAG-3, along with increased expression of transcription factors like thymocyte selection-associated HMG bOX (TOX; encoded by *TOX*). They also demonstrate reduced capacity for proliferation, decreased production of cytokines including IFN-γ, TNF-α, and IL-2, and impaired ability to kill tumor cells [179]. A recent single-cell RNA sequencing study identified key hub genes such as class I–restricted T cell–associated molecule (CRTAM; encoded by CRTAM), C-type lectin domain–containing 2D (CLEC2D; encoded by CLEC2D), and killer cell lectin-like receptor subfamily B member 1 (KLRB1; encoded by KLRB1) that are characteristic of exhausted CD8^+^ T cell states in breast cancer. These genes can be used to predict patient prognosis and their potential response to immunotherapy [179].

Clinically, the presence of exhausted CD8^+^ T lymphocytes that infiltrate tumors is associated with decreased overall survival of patients with ER^+^ BC but not in patients with TNBC. High intratumoral CD8^+^ T_EX signatures and IFNγ signaling were found to be associated with considerably poorer overall and relapse-free survival in premenopausal women in a study utilizing early-stage ER^+^ tumor cohorts. Beyond conventional Oncotype DX categorization, this analysis identified high-risk people [180]. Within the environment of breast tumors, exhausted CD8^+^ T cells play a role in weakening the immune response in several ways. For example, PD-1 levels are high on T cells that have entered the tumor, and these PD-1 molecules interact with PD-L1 found on both tumor cells and certain myeloid cells. This interaction sends inhibitory signals that block T cell activation and lower the production of IL-2, which results in the T cells becoming less responsive and less effective at fighting the cancer [181].

The transcription factor TOX is notably increased in PD-1 high CD8^+^ TILs, and it plays a key role in reinforcing the exhausted state of these cells at the epigenetic level. By maintaining TOX expression, the exhausted T cells keep up their inhibitory receptors and are prevented from developing into fully functional effector cells [181,182]. In a recent study involving breast cancer patients, researchers found that CD8^+^ T cells with high levels of PD-1 showed much higher levels of TOX compared to those with intermediate or no PD-1 expression. These cells also co-expressed exhaustion markers like CD39, TIGIT, and B and T lymphocyte attenuator (BTLA; encoded by *BTLA*), indicating they are likely terminally exhausted. Additionally, these PD-1 high cells produced significantly less IL-2, TNF, and IFN-γ when stimulated in *ex vivo* conditions, which confirms that their ability to function is impaired [183].

#### 3.3.4. γδ T cells

Although γδ T cells are generally recognized for their capacity to eliminate tumor cells, emerging research indicates that specific subsets of these cells in breast cancer can adopt a regulatory and immunosuppressive role. This function enables the tumor to escape immune detection and promotes metastasis. In mouse models of spontaneous breast cancer, tumor-derived IL-1β stimulates γδ T cells to produce IL-17, which in turn increases the expansion of neutrophils dependent on granulocyte colony-stimulating factor (G-CSF; encoded by *GCSF*). These neutrophils acquire a phenotype that suppresses CD8^+^ T cell activity and contributes to metastasis in the lungs and lymph nodes, even when primary tumor growth remains unaffected. Interventions that neutralize IL-17, G-CSF, or deplete γδ T cells significantly decrease the metastatic spread [184].

In human breast cancer tissues, a specific subset of Vδ1-positive γδ T cells that express CD73 has been identified. These cells also produce IL-10, interleukin-8 (IL-8; encoded by *IL8*), and PD-L1, and they possess strong immunosuppressive capabilities. When grown together with allogeneic CD4^+^ or CD8^+^ αβ T cells, these CD73-expressing Vδ1 cells notably suppress their proliferation and the production of effector cytokines such as IFN-γ, perforin, and granzyme B [185]. The presence of these regulatory γδ T cells within the tumor is associated with increased infiltration of FOXP3^+^ regulatory T cells and decreased presence of cytotoxic CD8^+^ T cells. This pattern is also linked to worse clinical outcomes in BC patients [186].

A recent investigation has indicated that BC cells exhibiting elevated levels of TIM3 are particularly prevalent during the initial phases of micro metastatic seeding. The study elucidates that TIM3 expression stimulates the activation of β-catenin and IL1β signaling pathways, which contribute to stem-like phenotype in cancer cells, their survival, and the recruitment of immunosuppressive γδ T cells within the micro metastatic niche. These γδ T cells play a role in inhibiting the infiltration and activity of local CD8^+^ T cells, thereby supporting metastatic progression. Notably, the presence of TIM3^+^ tumor cells correlates with a poorer prognosis in patients with breast cancer. From a therapeutic perspective, blocking TIM3 in preclinical models of micro metastasis resulted in a significant reduction of γδ T cell induction, an increase in CD8^+^ T cell infiltration, and a decrease in metastatic burden. These findings underscore TIM3’s role as a critical mediator of γδ T cell-driven immune suppression within the tumor microenvironment during metastasis [187].

#### 3.3.5. Myeloid-derived suppressor cells

MDSCs are a diverse group of immature myeloid cells that tend to accumulate in cases of breast cancer, where they play a significant role in suppressing the immune response. In human breast tumors, these MDSCs are typically characterized by the presence of CD11b^+^, CD33^+^, and low or absent human leukocyte antigen A class II histocompatibility antigen-DR (HLA-DR; encoded by *HLA-DRA* and *HLA-DRB*) expression. Their numbers tend to increase both systemically and within the tumor microenvironment, and their abundance has been associated with greater tumor burden and poorer prognosis [188]. MDSCs, which include both polymorphonuclear (PMN-MDSCs) and monocytic (M-MDSCs) subsets, increase significantly in breast cancer. They play a role in helping the tumor evade the immune system. Higher levels of MDSCs found in the blood and tumor tissues are associated with larger tumors, more advanced cancer stages, metastasis, and a poorer response to treatments, including ICIs [189]. In mouse models, two main types of MDSCs are identified: monocytic MDSCs (M-MDSCs), characterized by integrin alpha M-positive/lymphocyte antigen 6 family member C-high/lymphocyte antigen 6 family member G-negative (CD11b^+^Ly6C^high^Ly6G^−^; encoded by *CD11B, LY6C*, and *LY6G*) markers, and granulocytic MDSCs (PMN-MDSCs), characterized by CD11b^+^Ly6G^+^Ly6C^low^ markers. Both subsets play a role in actively suppressing the immune response against tumors [190]. M-MDSCs in breast cancer express arginase-1 (ARG1; encoded *ARG1*) and iNOS, which deplete L-arginine and produce nitric oxide, respectively. This impairs TCR ζ-chain expression in T cells, halts proliferation, and decreases effector function, ultimately weakening CD8^+^ T cell responses [191]. PMN-MDSCs generate ROS and peroxynitrite, which chemically alter the TCR complex. This modification can lead to the apoptosis of T cells and hinder their ability to recognize and respond to antigens, thus impairing immune function [192]. Breast cancer-associated MDSCs increase the expression of PD-L1 and release cytokines such as IL-10 and TGF-β. This process activates the PD-1 pathway on T cells, which in turn promotes the expansion of regulatory T cells and M2-like macrophages. These cells suppress the immune response by inhibiting adaptive immunity and impairing antigen presentation functions of dendritic cells and NK cells [193]. Breast tumors produce various factors, such as G-CSF, granulocyte/macrophage stimulating factor (GM-CSF), IL-6, IL-1β, IL-17, TGF-β, C-X-C motif chemokine 2 (CXCL2; encoded by *CXCL2*), C-X-C motif chemokine 5 (CXCL5; encoded by *CXCL5*), and CCL2. These substances facilitate the development and migration of MDSCs from the bone marrow to the tumor environment. In mouse models of mammary carcinoma, these cytokines support both the growth and functional activation of MDSCs [163].

## 4. Therapeutic Opportunities by Targeting Immune Evasion

It’s encouraging to see that immunotherapy is gaining significant attention and success in clinical settings. It is truly beneficial for patients if cancer eradication occurs through boosting their own immune system. The immense power and success of immunotherapy was recognized with the Nobel Prize in 2018 to James P. Allison and Tasuku Honjo, for their groundbreaking discovery related to negative immune regulation, specifically their work on immune checkpoint blockade. Following this, many successes have been achieved by scientists and clinicians through immune modulation and immunotherapy in cancer treatment. Here, we have listed some successful clinical studies in Table 2, which are linked to anti-immune evasion strategies against BC by targeting specific immune checkpoint candidates responsible for immune evasion.

## 5. Challenges and Future Directions

ICB has significantly improved the outcomes for a subset of patients with TNBC. However, it is unclear why only a subset responds to ICB, reflecting inter-patient heterogeneity [194]. Currently, PD-L1 expression, tumor mutation burden, and TILs are used to predict response in the clinic but have limited predictive power [195]. Understanding the molecular mechanisms and biomarkers predictive of response to ICB in TNBC, therefore, remains an open area of investigation. Imaging mass cytometric analysis showed that the top two predictors of ICB response at baseline were the proliferative fractions of MHCI & II^high^ cancer cells and CD8^+^TCF1^+^ T cells [195]. Moreover, cell phenotype and spatial organization are major predictors of ICB response in TNBC [196]. This significant intratumoral heterogeneity was further demonstrated by whole mounts on lumpectomies for breast cancer and quantitative multiplex immunofluorescence (MxIF) imaging. While most of the tumor cores represented a single molecular subtype, in 13 out of the 38 tumor cores, even within the same tumor, there were subtypes and immune niches, making heterogeneity a major problem in ICB response [197].

Recent studies have also focused on understanding how tumor heterogeneity and cellular plasticity affect immune evasion in breast cancer. While much of the work has focused on understanding mechanisms of immune evasion in established tumors, these mechanisms during tumor initiation are only beginning to be understood. A recent study showed that CXCR4^+^ niche macrophages regulate the tumor-initiating activity of various breast cancer subtypes by enhancing TIC survival and tumor-forming capacity, while promoting early immune evasion through regulatory T cell induction [198]. Understanding the changes in the immune niche and immune evasion in the early stages will be crucial for developing better prevention methods and predicting tumor progression.

Another open challenge for the breast cancer community remains to understand the impact of aging on mechanisms of immune evasion. BC risk increases with age, with most women diagnosed in the post-menopausal phase [199]. Aging induces significant changes in both the adaptive and innate immune system, skewing hematopoiesis toward myelopoiesis, while contracting lymphopoiesis [200,201]. We are still learning how age-related shifts in our immune system affect both the development of cancer and how patients respond to treatment. Early research on immunotherapy, like immune checkpoint blockade, shows that responses can differ significantly based on a patient’s age. For example, studies in TNBC have shown that there is an age-induced immunosenescence where TIL counts decreased linearly with patient age across different genetic TNBCs [202]. An additional factor affecting breast cancer patients is that with menopause, there is a significant decline in circulating estrogen [203]. Estrogen has previously been shown to affect both innate and adaptive immune cells, and estrogen can also directly alter the expression of immune evasion molecules [204]. However, most of the studies using syngeneic mouse models are done in young mice, and they are unable to directly assess the impact of aging on mechanisms of immune evasion. Gaining a deeper understanding of these age-specific responses in the future will be crucial for developing personalized, age-stratified therapies for BC patients in the future.

## Figures and Tables

**Table 1.** Comparison of TIME composition and characteristics in the 3 ‘intrinsic’ subtypes of BC: HR+, HER2+, and TNBC.

TIME Feature	Hormone receptor-positive (HR+)	Human epidermal growth factor-positive (HER2+)	Triple-negative breast cancer (TNBC)
CD8^+^ T cells	Low CD8^+^ cytotoxic T-lymphocyte infiltration; luminal tumors generally have the fewest CD8^+^ tumor infiltrating lymphocytes (TILs) among subtypes. Consequently, immune responses are weak in most HR+ luminal cancers [83].	Moderate CD8^+^ TIL levels; HER2+ tumors show significantly more CD8^+^ infiltration than HR+ luminal tumors. CD8^+^ T cells contribute to anti-HER2 immune effects in these tumors [74,84].	High CD8^+^ TIL density is typical, reflecting an immunogenic ‘hot’ microenvironment. Basal-like TNBC cancers have the greatest CD8^+^ infiltration, correlating with better prognosis when present [85].
CD4^+^ T cells	Helper T cells (Th1) are scarce, and HR+ luminal tumors often lack forkhead box P3-positive (FOXP3+) regulatory T cells entirely. Thus, the regulatory T-cell (Treg):CD8 ratio is generally low in luminal BC. When Tregs are present in HR+ tumors, a high Treg/CD8 ratio portends worse outcomes [86]	Th1-like activity is somewhat increased compared to luminal BC, and FOXP3^+^ Tregs infiltrate more readily (higher Treg numbers than in luminal). The Treg:CD8 ratio in HER2+ tumors is elevated relative to luminal tumors [86]	Enriched in both effector and regulatory CD4^+^ T cells, TNBC displays Th1-skewed inflammation (interferon gamma (IFNγ; encoded by IFNG)), with high Th1 chemokines, alongside abundant FOXP3^+^ Tregs. The Treg fraction is significantly higher than in luminal cancers, contributing to an immunosuppressive component within an overall inflamed milieu [79].
B cells	Often minimal infiltration by B lymphocytes. HR+ luminal tumors rarely develop tertiary lymphoid structures, so intratumoral B cells are uncommon. The density of all TIL subsets (including B cells) is consistently lower in luminal tumors [87]	HER2-driven tumors can harbor moderate B-cell/Tertiary lymphoid structures (TLS) infiltration, especially in immune-rich cases (e.g., some HER2+ tumors show brisk TILs including CD20^+^ cells). However, baseline B-cell infiltration is lower than in TNBC [88]	Frequently infiltrated by B cells, often organized in tertiary lymphoid structures. High-TIL TNBCs are enriched in CD20^+^ B cells and clonal IgG^+^ populations, reflecting robust humoral immune responses. The majority of TNBCs contain TLS with germinal centers, underscoring a prominent B-cell component [88]
Dendritic cells	Luminal tumors generally exhibit low numbers of conventional dendritic cells (DCs), limiting antigen presentation. Plasmacytoid DCs (pDC) are also sparse, contributing to the overall “cold” immune landscape [89]	HER2+ breast cancers can recruit both conventional/classical cDC subsets (cDC1 and cDC2), which are associated with intermediate levels of immune activation. pDC infiltration is variable. Overall, DC-mediated T-cell priming in HER2+ tumors is more robust than in luminal but less pronounced than in TNBC [90].	Enriched in dendritic cells, especially cDC1 which are preferentially recruited into TNBC lesions. Higher intratumoral cDC1 and pDC densities accompany the immune-inflamed phenotype of TNBC. Notably, pDC infiltration in TNBC correlates with TIL abundance and improved survival under therapy [89]
Macrophages (TAMs, M1/M2)	Tumor-associated macrophages are present but at relatively low levels. Luminal tumors tend to have fewer TAMs overall, though those present often exhibit an M2-like, pro-tumor phenotype (high CD163^+^ expression). ER^+^ status is associated with lower CD68/CD163 infiltration. When TAMs are abundant in luminal BC, they can foster immunosuppression and endocrine resistance [84]	HER2+ tumors accumulate high numbers of tumor-associated macrophages (TAMs), including PD-L1^+^ immunosuppressive macrophages. Both M1 and M2 polarization are observed, but immunosuppressive M2-like TAMs are particularly elevated in HER2+ cases (high CD163^+^ cell proportions). TAM-derived factors (e.g., C-C motif chemokine ligand 2 (CCL2; encoded by *CCL2)*) are implicated in HER2+ tumor progression [74,84].	Basal-like TNBCs contain abundant CD68^+^ and CD163^+^ macrophages, reflecting both M1-like (pro-inflammatory) and M2-like (immunosuppressive) populations in the tumor immune microenvironment (TIME). These TAMs can express PD-L1 and secrete cytokines that modulate TIL function. Notably, high TAM presence in TNBC is associated with aggressive features, but also with potential responsiveness to macrophage-targeted therapies [74,84]
Myeloid-derived suppressor cells (MDSCs)	Generally low levels of MDSC cells. HR+ luminal cancers, being less inflammatory, do not strongly recruit MDSCs. Circulating and intratumoral MDSC levels are typically lower than in more aggressive subtypes. However, in advanced luminal disease, tumor-secreted factors, such as interleukin-6 (IL-6; encoded by *IL6*), can induce some MDSC accumulation, contributing to immunosuppression [91]	HER2-driven tumors often produce pro-inflammatory cytokines (e.g., granulocyte colony-stimulating factor (G-CSF; encoded by *GCSF* and IL-6) that can expand MDSCs. MDSC infiltration in HER2+ BC is variable, but higher than in luminal A. Notably, anti-HER2 therapy resistance has been linked to MDSC-mediated immune evasion, indicating a role for these suppressive myeloid cells in HER2+ contexts [92].	TNBC tumors secrete chemokines, such as C-X-C motif chemokine 2 (CXCL2; encoded by CXCL2) and C-C motif chemokine ligand 22 (CCL22; encoded by CCL22), that robustly recruit MDSCs to tumors and metastatic sites. Accordingly, TNBC patients show significantly higher intratumoral and circulating MDSC counts than non-TNBC patients. These MDSCs (both polymorphonuclear and monocytic) potently suppress T-cell activity and can promote metastasis in TNBC [91].
Natural Killer (NK) cells	Poor NK cell infiltration. Luminal tumors typically exhibit very few tumor-infiltrating NK cells, partly due to low immunogenicity and possibly higher major histocompatibility complex class I (MHC-I) molecule expression on tumor cells (limiting NK activation). Overall, HR+ tumors are considered 'NK-cold' [93].	Moderate NK cell recruitment. HER2+ cancers attract NK cells, which are crucial effectors of antibody-dependent cellular cytotoxicity against HER2-targeted therapies. Untreated HER2+ tumors can have intermediate NK cell levels in the stroma. Upon trastuzumab treatment, NK cell infiltration and activation increase markedly. Baseline, however, HER2+ TIME is only mildly NK-enriched compared to TNBC [74,94]	Enriched in NK cells, though often functionally suppressed. TNBC tumors show higher baseline NK cell infiltration than luminal tumors, reflecting their immunogenic nature. However, many NK cells in TNBC are 'exhausted' or immature, as TNBC can downregulate NK-activating ligands. Nonetheless, the presence of intratumoral NK cells in TNBC is associated with improved immune responses and may synergize with immunotherapy [95]
Cancer-Associated Fibroblast Subtypes (CAF-S1/S2/S4)	Luminal tumors are rich in CAF-S2 subtype fibroblasts, which are relatively quiescent and not strongly immunomodulatory. They lack the myofibroblastic activation seen in more aggressive subtypes. CAF-S1 (the immunosuppressive, myofibroblastic CAF) is infrequent in luminal BC, aligning with the minimal TIL presence. Overall, luminal stroma is less desmoplastic and less immunologically active [74,96].	HER2^+^ breast tumors exhibit a heterogeneous composition of CAF subtypes. Some activation of CAF-S1 and CAF-S4 populations is observed, consistent with their high-grade nature, though typically less pronounced than in TNBC. The HER2^+^ stroma is often variably desmoplastic. Evidence suggests that CCL2-driven fibroblast–macrophage interactions contribute to tumor growth in this subtype, implicating CAF activity. However, the specific prevalence and functional roles of individual CAF subsets in HER2^+^ disease remain less well characterized compared to TNBC [97].	Enriched in immunomodulatory CAF subsets. TNBC exhibits accumulation of CAF-S1 and CAF-S4 myofibroblasts. CAF-S1 in TNBC actively creates an immunosuppressive niche by secreting C-X-C motif chemokine 12 (CXCL12; encoded by *CXCL12*) and checkpoint ligands, thereby recruiting and retaining Tregs. CAF-S4 also contributes to a stiff, desmoplastic stroma. The presence of CAF-S1/S4 in TNBC skews the Treg:CD8 balance toward immunosuppression. These fibroblast subtypes are far more prevalent in TNBC than in luminal disease [74].
Cytokines & Chemokines	Luminal breast tumors produce minimal IL-6 and typically do not activate signal transducer and activator of transcription 3 (STAT3; encoded by STAT3)–mediated inflammation. They also secrete lower levels of interferon gamma–inducible chemokines such as C-X-C motif chemokine ligand 9 (CXCL9; encoded by CXCL9) and C-X-C motif chemokine ligand 10 (CXCL10; encoded by CXCL10), which is consistent with weak Th1 activity. Instead, luminal microenvironments might have transforming growth factor beta (TGF-β; encoded by *TGFB1*)–driven signaling and interleukin-10 (IL-10; encoded by *IL10*) produced by a few M2 macrophages, contributing to immune exclusion. In general, HR+ tumors tend to have an anti-inflammatory cytokine environment (like TGF-β) and don’t have strong proinflammatory signals [98,99].	Overexpression of HER2+ causes a strong IL-6 autocrine loop, which activates STAT3 and causes inflammation throughout the body. HER2+ tumors frequently exhibit elevated levels of IL-6 and IL-8, which facilitate cancer stemness and the recruitment of myeloid cells. They also make chemicals like CXCL13 which attract T and B cells to them. The activation of TGF-β is moderate; notably, non-luminal breast cancers, including human epidermal growth factor receptor 2-enriched (HER2-E) subtypes, exhibit elevated TGF-β signaling compared to luminal subtypes [99]	TNBC tumors release a lot of proinflammatory cytokines, and IL-6 and IL-8 are usually very high. These cytokines are important for TNBC cells to survive and spread. They also make a lot of IFNγ-induced chemokines, such as CXCL9/CXCL10, which is linked to strong Th1/Tc1 recruitment. TNBC, particularly mesenchymal subtypes, can paradoxically activate robust TGF-β pathways, with basal-like cancers exhibiting the most pronounced TGF-β response among subtypes. Additionally, TNBC's M2-TAM and regulatory B/T cells secrete IL-10, which suppresses the immune system. So, the cytokine environment in TNBC is a mix of proinflammatory signals that bring in TILs and counter-regulatory cytokines like IL-10 and TGF-β that can stop the immune system from attacking [99].
Immune Checkpoints	Low checkpoint expression – an ‘immune-desert’ profile. Luminal-A cancers have the lowest prevalence of PD-L1 expression on tumor/immune cells [100].	Moderate checkpoint upregulation. HER2+ tumors show higher PD-L1 positivity on tumor or immune cells, significantly more than luminal BC [100].	Checkpoint-high, immune-evasive phenotype. TNBCs have the highest PD-L1 expression rates, mainly in the basal-like subset. PD-L1 is expressed on both tumor cells and infiltrating myeloid cells, contributing to immune suppression [100].

**Table 2.** Therapeutic Strategies Targeting Immune Evasion.

Target	Treatment	Breast Cancer Indication	Strategy	Clinical Trial Study Status & Phase	NCT Number
PD-1	Pembrolizumab + chemotherapy	Metastatic TNBC	PD-1 antibody + Nab-Paclitaxel/Paclitaxel/Gemcitabine+Carboplatin	Completed, Phase 3	NCT02819518
	Nivolumab + chemotherapy	Luminal B w/ basal molecular subtype	PD-1 antibody + triptorelin + exemestane + Epirubicin and Cyclophosphamide	Completed, Phase 2	NCT04659551
	Nivolumab + chemotherapy	ER+/HER2−	PD-1 antibody + paclitaxel + anthracycline + cyclophosphamide	Completed, Phase 3	NCT04109066
PD-1 × microtubule	Pembrolizumab + Eribulin Mesylate	Metastatic HR+	PD-1 antibody + microtubule inhibitor	Completed, Phase 2	NCT03051659
	Pembrolizumab + Eribulin Mesylate	Metastatic TNBC	PD-1 antibody + microtubule inhibitor	Completed, Phase 1b/2	NCT02513472
PD-1 × tyrosine kinase	Nivolumab + Cabozantinib (XL184)	Metastatic TNBC	PD-1 antibody + VEGFR/MET inhibitor	Completed, Phase 2	NCT03316586
PD-1 × tumors	Pembrolizumab + CyPep-1	Advanced metastatic cancer (incl. breast)	PD-1 antibody + oncolytic chemically synthesized peptide	Completed, Phase 1b/2a	NCT05383170
PD-L1	Atezolizumab + chemotherapy	Metastatic TNBC	PD-L1 antibody + Nab-Paclitaxel	Completed, Phase 3	NCT03125902
	Durvalumab + chemotherapy	TNBC	PD-L1 antibody + Nab-paclitaxel + Epirubicin and Cyclophosphamide	Completed, Phase 2	NCT02685059
	Atezolizumab + chemotherapy	TNBC	PD-L1 antibody + carboplatin and nab-paclitaxel	Completed, Phase 3	NCT02620280
PD-L1 × tumors	Atezolizumab + Talimogene Laherparepvec	TNBC	PD-L1 antibody + oncolytic virus	Completed, Phase 1b	NCT03256344
CTLA-4 × PD-1	INT230-6 + pembrolizumab + ipilimumab	Advanced refractory cancers (incl. breast)	(cell permeation enchancer + cisplatin + vinblastine sulfate) + PD-1 antibody + CTLA-4 antibody	Completed, Phase 1/2	NCT03058289
	NKTR-214 + nivolumab + ipilimumab	TNBC and HR+/HER2−	recombinant human interleukin 2 + PD-1 antibody + CTLA-4 antibody	Completed, Phase 1/2	NCT02983045
CTLA-4 × PD-L1	tremelimumab + durvalumab + chemotherapy	HR+/HER2−	CTLA-4 antibody + PD-L1 antibody + standard NACT	Completed, Early Phase 1	NCT03132467
	tremelimumab + durvalumab	Metastatic HER2−	Anti-CTLA-4 antibody + PD-L1 antibody	Completed, Phase 2	NCT02536794
CTLA-4 × PD-L1 × TLR3	poly-ICLC + tremelimumab + durvalumab	Locally recurrent or metastatic breast cancer	TLR3 agonist + CTLA-4 antibody + PD-L1 antibody	Completed, Phase 1/2	NCT02643303
CTLA-4 × PD-L1 × microtubules	Metronomic Oral Vinorelbine + Durvalumab + Tremelimumab	Advanced refractory cancers (incl. breast)	Microtubule inhibitor + PD-L1 antibody + CTLA-4 antibody	Completed, Phase 1/2	NCT03518606
CTLA-4 × PD-L1 × HER2	KN026 + KN046	HER2+ solid tumors	Antibody bispecific to HER2 epitopes + CTLA-4/PD-L1 bispecific antibody	Completed, Phase 1	NCT04040699
LAG-3 × CTLA-4 × PD-1	XmAb^®^22841 + pembrolizumab	TNBC	LAG-3 × CTLA-4 bispecific antibody + PD-1 antibody	Completed, Phase 1	NCT03849469
MHC Class II (via LAG-3-Fc fusion)	IMP321 + chemotherapy	Metastatic HR+	APC stimulator via MHC-II to boost T cell priming + paclitaxel	Completed, Phase 2	NCT02614833

## Abbreviations

BC: Breast cancer
HR+: Hormone receptor-positive
HER2+: Human epidermal growth factor receptor 2-positive
TNBC: Triple-negative breast cancer
TIME: Tumor immune microenvironment
TAMs: Tumor-associated macrophages
DC: Dendritic cells
NK: Natural killer
CTL: CD8^+^ cytotoxic T lymphocytes
MDSCs: Myeloid-derived suppressor cells
Tregs: regulatory T cells
MHC-I: major histocompatibility complex class I
T_EX: T cell exhaustion
TILs: tumor-infiltrating lymphocytes
TLSs: Tertiary Lymphoid Structures
Tfhs: T follicular helper cells
PD-L1: Programmed death-ligand 1
CTLA-4: Cytotoxic T-lymphocyte-associated protein 4
LAG-3: Lymphocyte activating-3
PD-1: programmed cell death protein-1
iNOS: Inducible nitric oxide synthase
Bregs: Regulatory B cells
scRNA-seq: Single-cell RNA sequencing
TCF1^+^: T-cell factor 1-positive
DCIS: Ductal carcinoma *in situ*
IDC: Invasive ductal carcinoma
CCL2^+^: Chemokine ligand 2-positive
NAC: Neoadjuvant chemotherapy
EMT: Epithelial-to-mesenchymal
ICB: Immune checkpoint blockade
TICs: Tumor initiating cells
CSCs: Cancer stem cells
ICIs: Immune checkpoint inhibitors
ADCC: Antibody-dependent cellular cytotoxicity
sEVs: Small extracellular vesicles
SASP: Senescence-associated secretory phenotype
AP-1: Activator protein 1
METABRIC: Molecular Taxonomy of Breast Cancer International Consortium
VISTA: V-domain Ig suppressor of T cell activation
ALDH-2: Aldehyde dehydrogenase 2
DX: Diagnosis
HMG: High mobility group box
TME: Tumor microenvironment
